# Activation of creER recombinase in the mouse calvaria induces local recombination without effects on distant skeletal segments

**DOI:** 10.1038/s41598-021-87611-2

**Published:** 2021-04-15

**Authors:** Jue Hou, Charles P. Lin, Giuseppe Intini

**Affiliations:** 1 Advanced Microscopy Program, Center for Systems Biology and Wellman Center for Photomedicine, Massachusetts General Hospital, Harvard Medical School, Boston, MA 02114 USA; 2 Department of Periodontics and Preventive Dentistry, University of Pittsburgh School of Dental Medicine, Pittsburgh, PA USA; 3 Center for Craniofacial Regeneration, University of Pittsburgh School of Dental Medicine, Pittsburgh, PA USA; 4 University of Pittsburgh McGowan Institute for Regenerative Medicine, Pittsburgh, PA USA; 5 University of Pittsburgh UPMC Hillman Cancer Center, Pittsburgh, PA USA

**Keywords:** Biological models, Experimental organisms, Mesenchymal stem cells

## Abstract

Conditional creER-mediated gene inactivation or gene induction has emerged as a robust tool for studying gene functions in mouse models of tissue development, homeostasis, and regeneration. Here, we present a method to conditionally induce cre recombination in the mouse calvarial bone while avoiding systemic recombination in distal bones. To test our method, we utilized Prx1creER-egfp;td-Tomato mice and delivered 4-hydroxytamoxifen (4-OHT) to the mouse calvaria, subperiosteally. First, we showed that two calvaria subperiosteal injections of 10 µg of 4-OHT (3.3 mg of 4-OHT/kg of body weight) can induce local recombination as efficiently as two intraperitoneal systemic injections of 200 μg of tamoxifen (70 mg of tamoxifen/kg of body weight). Then, we studied the recombination efficiency of various subperiosteal calvaria dosages and found that two subperiosteal injections of 5 µg 4-OHT (1.65 mg of 4-OHT/kg of body weight) uphold the same recombination efficiency observed with higher dosages. Importantly, the result indicated that the low dosage does not induce significant systemic recombination in remote skeletal tissues. With the proposed local low dosage protocol, the recombination efficiency at the injection site (calvarial bone) reached 94%, while the recombination efficiency at the mandible and the digits was as low as the efficiency measured in control animals.

## Introduction

Cre recombinase is a powerful and popular gene editing tool utilized in vitro and in vivo to inactivate or induce expression of genes or molecular markers^1^. The expression of cre recombinase can be further controlled by tissue-specific/cell-specific promoter or enhancer sequences to induce gene expression or suppression in specific tissues and cell lineages. The high gene editing efficiency and simplicity of the cre recombinase-based methods enable a broad spectrum of studies related to gene functions, cell functions, cell lineage tracing, or organ functions.

In order to have a better spatial and temporal control of its activity, the cre recombinase has been modified with the fusion of a modified estrogen receptor and a chaperone protein^2^. The so obtained tamoxifen-dependent inducible construct is referred to as creER, where ER stands for the mutated hormone-binding domain of the estrogen receptor. In presence of agonists of the modified estrogen receptor, such as tamoxifen, the interaction of the creER recombinase and the chaperone protein is disrupted, allowing the nuclear translocation of the creER and the subsequent recombination at the loxP sites^3^. To achieve high recombination efficiency tamoxifen is given systemically to the animals through intraperitoneal (IP) injection or it is incorporated into their daily diet or water^4^. However, it is the tamoxifen metabolites (4-hydroxytamoxifen and endoxifen), rather than tamoxifen itself, that enables the creER-loxP recombinase in all the tissues or cells where creER is expressed^5^. Consequently, both tamoxifen and 4-OHT can be used to induce systematic recombination with IP injection, and tamoxifen is preferred in most induction protocols due to its lower cost^6–8^. However, when the recombination is induced locally, the metabolites should be preferred since local delivery of tamoxifen would require systemic metabolic processing, resulting in lower local recombination efficiency^9–11^. Importantly, incomplete and unspecific recombination is still reported, even with the use of local delivery of 4-OHT, as creER activity is often observed outside of the treated sites and organs^12–14^. For instance, Seime and et al. observed significant systemic recombination in bones of animals injected intramuscularly with 4-OHT^15^. Thus, the recombination efficiency of locally delivered ER agonists needs to be validated in an animal and tissue specific manner.

Craniofacial biologists and, more in general, bone biologists and tissue engineers, frequently refer to the mouse calvarial bones to perform experimental assays, since the easy surgical access to these bone facilitates the execution of the experiments^16,17^ while their flatness enables the in vivo microscopy evaluation^18^. A proper use of the creER technology in calvarial bone studies, with the ability to specifically activate creER locally rather than systemically, would facilitate the correct execution of studies aimed at evaluating the effects of local inactivation or activation of certain genes or molecular markers in the bone tissue. The ability to do so is significant, given the recent highlights on the skeleton as an organ with systemic interplays with other tissues, including muscles^19^, immune cells^20^, or even the central nervous system^21^.

Utilizing a transgenic mouse expressing creER under the control of the promoter of Paired-related homeobox 1 (Prx1 or Prrx1), a transcription factor expressed in limbs and craniofacial bones during embryogenesis, post-natal growth, and post-natal bone regeneration^17,22–26^, here we validate a method to induce local cre-mediated recombination in calvarial bone by means of subperiosteal delivery of 4-OHT. We tested the effects of local delivery of 4-OHT in post-natal skeletal tissues using Prx1-creER-eGFP;td-Tomato animals and measured the recombination efficiency by means of expression of the tdTOMATO fluorescent protein in Prx1 expressing cells. Specifically, we hypothesized that a proper regimen of calvarial subperiosteal delivery of 4-OHT can induce cre recombination in the Prx1 expressing cells of the calvarial bone, sparing recombination in Prx1 expressing cells of more remote bones, such as the mandible or the digit bones.

## Material and methods

### Animals

All procedures involving the use of animals were performed in compliance with the ARRIVE guidelines for animal studies and in compliance with institutional guidelines as approved by the Institutional Animal Care and Use Committee (IACUC) at Massachusetts General Hospital (IACUC approval 2015N000098). To evaluate the recombination efficiency of local subperiosteal injection of 4-OHT, we crossed Prx1-creER-eGFP^+/+^ (Jackson stock number 029211, genetic background C57BL/6 × SJL) male mice with tdTomato^+/+^ female mice carrying a loxP-flanked STOP cassette preventing transcription of a CAG promoter-driven tdTomato fluorescent protein (Jackson stock number 007914, genetic background C57BL/6) to obtain Prx1-creER-eGFP^+/−^;td-Tomato^+/−^ mice (hereafter, Prx1-creER-eGFP;td-Tomato). Genotyping was utilized to confirm the presence of both the prx1-creER-eGFP and the floxed tdTomato transgenes in the Prx1-creER-eGFP;td-Tomato mice. Since the creER is sensitive, although minimally, to endogenous estrogen^27^, only male mice were utilized throughout the study, so to avoid the recombination that may have been caused by the estrogen in female mice. Five-week old mice were utilized to maximize the chances of visualization and quantification of the fluorescent proteins during the active proliferation and differentiation stages of the early post-natal development.

To test the recombination efficiency of 4-OHT local injection in comparison to tamoxifen systemic IP injection, 12 5-day old mice (~ 3 g of weight) were randomly divided into 3 groups: (1) mice treated with 2 doses of 4% ethanol with calvarial subperiosteal injections (20 µl volume, 48 h interval between doses) (negative control); (2) mice treated with 2 doses of 10 µg of 4-OHT in 4% ethanol with calvarial subperiosteal injections (3.3 mg of 4-OHT/kg of body weight, 20 µl volume, 48 h interval between doses); or (3) mice treated with 2 doses of 200 µg of tamoxifen in corn oil with systemic IP injections (70 mg of tamoxifen/kg of body weight, 10 µl volume, 48 h interval between doses) (positive control).

To identify the minimally effective dosage of subperiosteal delivery of 4-OHT, 16 5-day old pups (~ 3 g of weight) were randomly divided into 4 groups: (1) mice treated with one dose of 5 µg of 4-OHT in 4% ethanol with a local calvarial subperiosteal injection (1.65 mg of 4-OHT/kg of body weight, 10 µl volume); (2) mice treated with two doses of 5 µg of 4-OHT in 4% ethanol with local calvarial subperiosteal injections (1.65 mg of 4-OHT/kg of body weight, 10 µl volume, 48 h interval between doses); (3) mice treated with one dose of 10 µg of 4-OHT in 4% ethanol with a local calvarial subperiosteal injection (3.3 mg of 4-OHT/kg of body weight, 20 µl volume); and (4) mice treated with two doses of 10 µg of 4-OHT in 4% ethanol with local calvarial subperiosteal injections (3.3 mg of 4-OHT/kg of body weight, 20 µl volume, 48 h interval between doses).

Calvarial bones, mandibular bones, and digit bones were imaged using previously describe protocols with our intravital microscope, 2 days after the last injection of tamoxifen or 4-OHT^28^.

### Reagents

Following the manufacturer recommendations, Tamoxifen (T5648, Sigma) and 4-OHT (H7904, Sigma) were first dissolved in sterile corn oil and in 100% ethanol respectively, to make 10 mg/ml stock solutions. The solutions were then aliquoted into 1.5 ml amber tubes and stored at − 20 °C. On the day of the experiment, the solutions were thawed, and final concentrations were prepared using sterile corn oil for Tamoxifen and 4% ethanol (in PBS) for 4-OHT. Immediately before injections, the solutions were warmed up in a 37 °C water bath. No side effects relevant to the studies performed have been previously reported for the two utilized solvents when delivered by means of IP or subperiosteal injections.

### Injection of tamoxifen and 4-OHT

For systemic delivery, the tamoxifen solution was injected intraperitoneally using a 31G needle at P5 and P7. For local calvarial injections we followed our previously validated method which ensures subperiosteal delivery of the injected reagent^17^. Briefly, a 31 G needle was gently inserted perpendicularly to the skull surface, stopping as soon as the needle touched the skull. Then, the 4-OHT solution was injected. To avoid systemic recombination that may occur via bloodstream spreading of the solution, if bleeding was observed during the subperiosteal injection animals were excluded from the study.

### In vivo fluorescence microscopy

The recombination efficiency was evaluated utilizing our custom-built intravital microscope^29^. Briefly, while imaging, the animals were maintained under anesthesia with 2% vaporized isofluorane and secured in a stable position under the microscope. The calvarial bone was surgically exposed for *en face* imaging. A femtosecond laser beam at 900 nm was focused on the region of interest through a 60× water immersion objective (LUMPLFLN60XW, Olympus). The second harmonic generation (SHG) from the bone and the fluorescence signals from eGFP and tdTomato were collected and separated with proper dichroic mirrors. The signals were detected with photomultiplier tubes with bandpass filters in the front (415–455 nm, 500–550 nm and 605–650 nm for SHG, eGFP and tdTomato respectively). At each imaging area, a 50 µm stack was taken with 1 µm step size.

### Image processing and automatic cell counting

**Figure 1:**
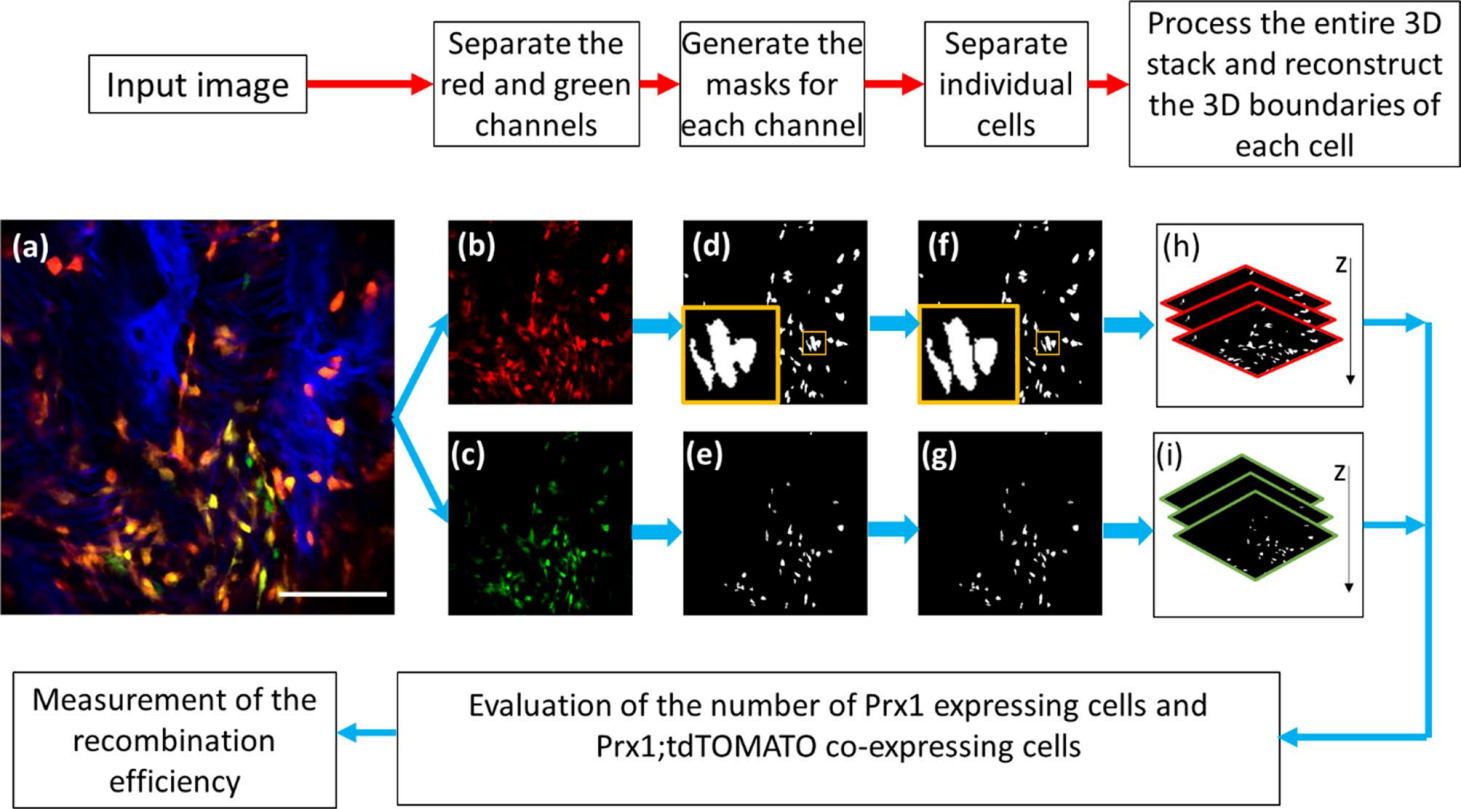
Image processing and automatic cell counting workflow. (**a**) A representative input image uploaded into Matlab program. After acquisition, the input image is processed to separate the red channel (**b**) and the green channel (**c**), which represent the cells that express tdTomato and Prx1 (eGFP) respectively. Subsequently, a three-component Otsu thresholding is independently applied to each channel, to generate a mask for tdTomato expressing cells (**d**) and Prx1 (eGFP) expressing cells (**e**). A marker-controlled watershed algorithm is applied to further separate the connected tdTomato expressing cells (**f**) and Prx1 (eGFP) expressing cells (**g**) into individual cells. The orange insets in (**d**) and (**f**) show connected cells (in **d**) that are successfully separated (in **f**). Then, 3D reconstructed masks for the tdTomato expressing cells (**h**) and for the Prx1 (eGFP) expressing cells (**i**) are generated to calculate the final recombination efficiency.

The multi-channel Z-stack images obtained during the in vivo imaging sessions were analyzed with a custom written Matlab program (Fig. 1). Briefly, to reduce noise signaling the images that captured eGFP and tdTomato were first preprocessed with a Gaussian filter with kernel size 5 × 5 (Fig. 1a–c). Then, the fluorescence signal was separated from the background noise by means of a three-component Otsu thresholding algorithm (Fig. 1d,e). The cleaned-up fluorescent images were then utilized to identify the local maximum signal in each cell using the Matlab built-in “regional maximum” function. Subsequently, the marker-controlled watershed algorithm was used to separate each Prx1-creER-eGFP and tdTomato expressing cell (Fig. 1f,g)^30^. The boundaries of the Prx1 expressing cells were identified using the Matlab built-in function “regionprops” and the number of tdTomato positive pixels were quantified inside each boundary (Fig. 1h,i). The Prx1+ cells were categorized as eGFP and tdTomato co-expressing cells if more than 50% of the eGFP pixels were also tdTomato positive, otherwise they were counted as Prx1 eGFP-expressing only cells. Cells expressing only tdTomato were not considered, as they would represent the progeny of Prx1+ cells (no longer expressing Prx1). The recombination efficiency was defined as the number of eGFP and tdTomato co-expressing cells (eGFP+;tdTomato+ cells) over the number of all eGFP+ cells.

### Statistics

Five regions of interest were randomly imaged for each suture or digit, in each animal. Data obtained from all the regions of interest in the animals of the same group was pooled and statistical analysis was performed using one-way analysis of variance to assess differences among groups. Statistically significant difference is indicated as *(p < 0.05). The results are presented as mean ± standard deviation.

## Results

**Figure 2:**
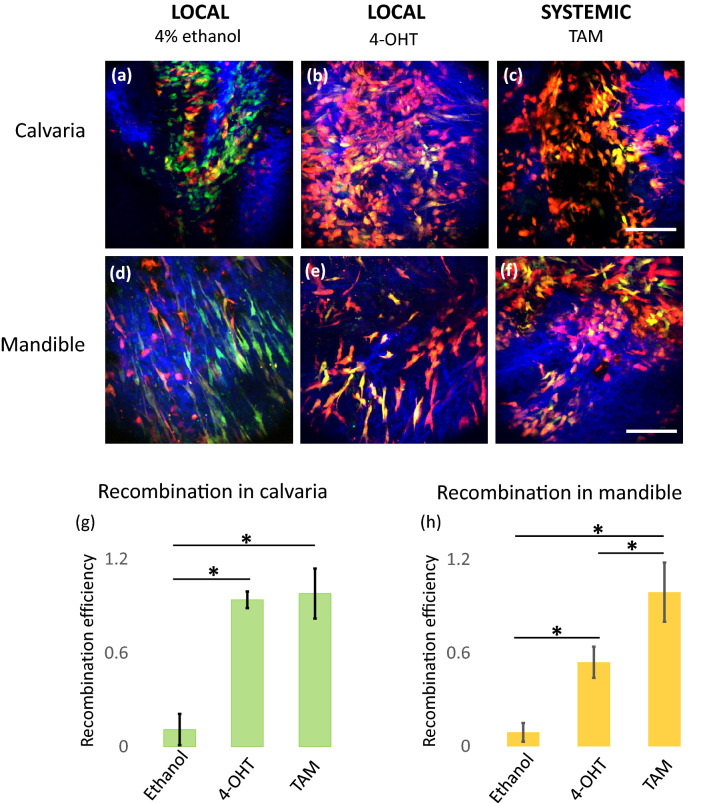
Comparison of recombination efficiency between locally induced and systemically induced recombination. Animals were treated with 4% ethanol (subperiosteal local injection) or 10 µg of 4-OHT (subperiosteal local injection) or 200 µg of Tamoxifen (systemic intraperitoneal (IP) injection). Representative images of the animal’s calvaria and mandibular bones are displayed: (**a**) and (**d**) 4% ethanol local injection, (**b**) and (**e**) 10 µg 4-OHT local injection, and (**c**) and (**f**) IP injections of 200 µg Tamoxifen (TAM). The Prx1 (eGFP) expressing cells are shown in green, the tdTomato expressing cells are shown in red and, thus, the eGFP and tdTomato co-expressing cells are shown in yellow. Bone is visualized with second harmonic generation and is pseudo-colored in blue. The scale bar represents 100 µm. The recombination efficiency measured in the different treatment groups is shown in histograms reporting the data collected in the animals’ calvaria (**g**) and the data collected in the animals’ mandibles (**h**). The error bars indicate standard deviations and the * indicates statistically significant difference with a p value < 0.05 (n = 4).

To evaluate whether local delivery of 4-OHT in the subperiosteum of the calvarial could effectively induce local cre recombination, we compared the recombination efficiency of local injection of 4-OHT to systemic IP injection of tamoxifen in Prx1-creER-eGFP;tdTomato mice (Fig. 2). Recombination efficiency was expressed in percentage (%) and was calculated counting the number of eGFP expressing cells that co-expressed tdTomato (eGFP+;tdTomato+ cells) over the number of all eGFP+ expressing cells. We observed that 11% of Prx1+ cells (eGFP expressing cells) co-expressed tdTomato in the animals that received 2 doses of 4% ethanol by local injections (Fig. 2a). This is the level of background non-specific recombination that may be considered as a “recombination leakage” in untreated mice. Differently, as expected, 98% of the Prx1+ cells were also tdTomato+ in the animals that received IP injection of 200 µg of tamoxifen. Significantly, the animals that received local subperiosteal injections of 4-OHT demonstrated recombination efficiency similar to the one observed in the animals that received IP injections of tamoxifen (99%). The recombination efficiency in the groups that received either 4-OHT local injections or tamoxifen IP injections was ~ 9 times higher than the one observed in the animals that were injected with 4% ethanol (p < 0.05), indicating that the local injection of 4-OHT was as efficient as IP injection of tamoxifen (Fig. 2g). The recombination efficiency measured in the mandibles of the animals that received 4-OHT, representing bones close to the area of local delivery, was significantly lower (54%) than the efficiency measured in the mandibles of animals that received tamoxifen IP injection (99%), but still 3 times higher than the efficiency observed in the mandibles of the animals that received 4% ethanol (17%) (Fig. 2h). All 12 animals involved in the study survived the treatment indicating a good tolerance of the current 4-OHT dosage.

**Figure 3:**
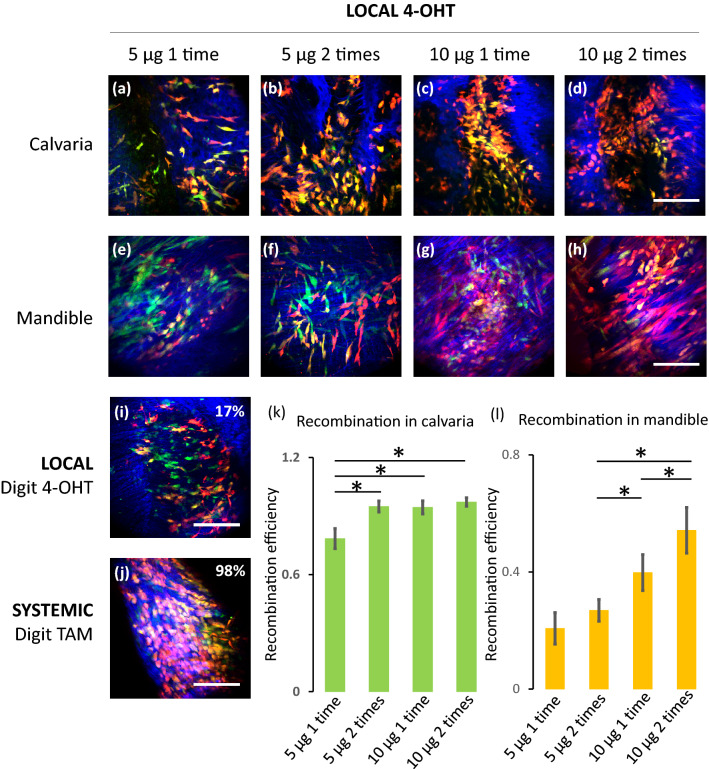
Recombination efficiency in animals treated with different local injection protocols. Representative images are displayed: (**a**) and (**e**) 5 µg 4-OHT local injection for one time in calvaria and mandible; (**b**) and (**f**) 5 µg 4-OHT local injections for two times in calvaria and mandible; (**c**) and (**g**) 10 µg 4-OHT local injection for one time in calvaria and mandible; and (**d**) and (**h**) 10 µg 4-OHT local injections for two times in calvaria and mandible. (**i**) Digit bone of a mouse treated with two local injections of 5 µg 4-OHT. (**j**) Digit bone of a mouse treated with two systemic IP injections of 200 µg of Tamoxifen. The Prx1 (eGFP) expressing cells are shown in green, the tdTomato expressing cells are shown in red and, thus, the eGFP and tdTOMATO co-expressing cells are shown in yellow. Bone is visualized with second harmonic generation and is pseudo-colored in blue. The scale bar represents 100 µm. The recombination efficiency measured in the different treatment groups is shown in (**i**) and (**j**) for the digits (inset numbers) and in histograms for the calvaria (**k**) and the mandibles (**l**). The error bars indicate standard deviations and the * indicates statistically significant difference with a p value < 0.05 (n = 4).

To test whether different dosages of local calvarial subperiosteal injection of 4-OHT can reduce the level of unwanted recombination in the mandible, we injected 4 groups of mice with 5 µg or 10 µg of 4-OHT for either 1 time or for 2 times with an interval of 48 h. The animals in all four treatment groups demonstrated good tolerance of the 4-OHT injection with only 1 animal that received 1 dose of 10 µg local 4-OHT dying during treatment. Animals that received a single dose of 5 µg presented with a 78% recombination efficiency in the calvaria and had the lowest recombination rate in the mandible (21%) (Fig. 3a,e,k,l). The other 3 groups (2 times 5 µg 4-OHT, 1 time 10 µg 4-OHT and 2 times 10 µg 4-OHT local injections) demonstrated more than 90% recombination efficiency in the calvaria (Fig. 3b–d,k). The level of recombination leakage observed in the mandible of animals that received one dose of 5 µg of 4-OHT is comparable to the one observed in the animals that received 2 doses of 5 µg of 4-OHT (p = 0.12) (Fig. 3e,f,l), and is 50% lower than that observed in the animals that received 2 doses of 10 µg of 4-OHT (p < 0.005) (Fig. 3e–h,l).

Finally, to test whether recombination leakage occurs in bones distant from the craniofacial bones, we evaluated the recombination efficiency in the middle digits of the animals' left hind legs that received 5 µg 4-OHT local injection for 2 times versus the ones that received tamoxifen IP (systemic). The recombination efficiency in the digits of the 4-OHT treated mice was much lower than the one measured in mice that received tamoxifen IP injection (p < 0.005) and was comparable to the baseline recombination observed in the control animals receiving 4% ethanol (p = 0.21) (Fig. 3i,j).

## Discussion

The development of the creER-loxP mouse models enabled experimental designs with spatial and temporal control of gene expression or gene inactivation^31–33^. For instance, by means of a promoter of choice to control the expression of the creER recombinase in tissues and cells, and by regulating the time of animal exposure to tamoxifen, the contribution of certain cellular mechanisms in specific cell populations at selected times can be evaluated^34–37^. However, a systemic treatment with tamoxifen, by inducing recombination in all cells and tissues of the animal body expressing the creER, represents a barrier to studies aiming at evaluating the effects of local modifications of gene expression. To overcome this barrier, researchers investigated the possibility of controlling the recombination with local administration of tamoxifen metabolites, such as 4-OHT, which can induce creER immediately and in loco. For instance, topical administration of 4-OHT has been proven to be effective in inducing recombination in the area where the chemical was applied. However, it was observed that the recombination extended in tissues distant from the application sites, and it was speculated that this systemic effect was due to the mice licking the areas of topical administration of 4-OHT^38,39^. Zadelaar et al*.* developed a poly-caprolactone-based perivascular delivering device that was implanted around the vessel of interest to slowly release 4-OHT^40^. Since this local delivering method requires a surgical implantation of the devise, the tissue damage and the local inflammation associated to the surgical procedure can alter the delivering devise and, consequently, the availability of the 4-OHT. Additionally, it is possible that some 4-OHT can enter the systemic circulation during implantation of the devise. To overcome these problems, a light activable creER recombinase was developed by placing the creER under the control of Eomes genomic locus^41^. However, as per all optical methods, this method is limited by the low penetration depth of the light. Thus, significant limitations exist in studies utilizing local activation of creER.

Skeletal tissue extends throughout the body and therefore studies that aim at controlling the local activation of creER in certain bone cells or bone segments may present with the same limitations discussed above, de facto impairing the ability to distinguish between a local and a systemic effect of the recombination. Here we provide a technical protocol that allows for the use of the creER-loxP mouse model to perform bone biology studies in the calvarial bone, allowing for a maximal locally induced cre recombination with a minimal systemically induced cre recombination. The protocol is based on a subperiosteal delivery of 4-OHT to induce cre recombination in the Prx1 expressing cells of the calvarial bone, avoiding significant levels of recombination in other craniofacial bones and in more distant bones, such as limb bones. To define the protocol, we first confirmed that calvarial local subperiosteal injection of 4-OHT can induce creER recombination as efficiently as the standard systemic IP injection of tamoxifen. Then, we further tested different local injection dosages and identified that two local injections of 5 µg 4-OHT with an interval of 48 h provided high recombination efficiency in the injection site, while limiting the unwanted recombination in other sites.

The described protocol presents with some limitations. For instance, it has been reported that the recombination efficiency of creER is strongly influenced by the pharmacokinetic profile of 4-OHT administration as the animals’ sex, age, injection site, and gene expression level play a role in the creER recombination efficiency^42^. Thus, while we proved that our injection protocol was successful at maintaining high recombination efficiency at the injection site while limiting leakage into distal bones, mice of different ages, sex, and strain may still present with different recombination activity, both locally and remotely. In addition, the possibility that different cre recombinase drivers (i.e., promoters, enhancers, etc.) can influence the expression and, consequently, the local and systemic activation of the recombinase needs to be further evaluated. Yet, the described protocol allows for a cre recombinase-based local analysis of the role that Prx1 expressing osteoprogenitor cells may have in bone homeostasis, bone diseases, and bone repair/regeneration without the risk that an unwanted systemic induced recombination may confound the results. Importantly, since it is well described that 4-OHT is toxic and the survival of the pups following its injection can be unpredictable^43^, our protocol also describes a method to minimize the exposure of mice to 4-OHT. Generally, the higher the dosage the higher the mortality rate and frequent injections can further induce damage to the treated organ/tissue^44,45^. Using small easily injectable reagent volumes, our protocol defines a low dosage treatment with minimal mortality rate (1/21). In our experience, this low mortality rate is comparable to the one observed in adult mice.

In conclusion, the described subperiosteal injection method provides a valuable tool for bone biology studies aimed at evaluating the local effects of certain gene manipulations in cells of the calvaria expressing Prx1. Since Prx1 is emerging as a valid marker of skeletal progenitor cells/skeletal stem cells^17,24,29,46–56^, this method provides the opportunity to study the role of these important cells and their molecular signaling by manipulating their gene expression in a local environment, with the significant confidence that the genetic manipulation do not occur systemically.
